# Tumor size, treatment patterns, and survival in neuro-oncology patients before and during the COVID-19 pandemic

**DOI:** 10.1007/s10143-023-02132-y

**Published:** 2023-09-06

**Authors:** Lydia Karamani, Aaron Lawson McLean, Marcel A. Kamp, Thomas E. Mayer, Wolf Müller, Nazife Dinc, Christian Senft

**Affiliations:** 1 Department of Neurosurgery, Jena University Hospital, Friedrich Schiller University, Am Klinikum 1, 07747 Jena, Germany; 2 Center for Neuro-Oncology, Jena University Hospital, Jena, Germany; 3 Comprehensive Cancer Center Central Germany, Leipzig, Germany; 4 Institute of Diagnostic and Interventional Radiology, Neuroradiology Section, Jena University Hospital, FriedrichSchillerUniversity, Jena, Germany; 5 Paul Flechsig Institute of Neuropathology, University Medicine Leipzig, Leipzig, Germany

**Keywords:** COVID-19, Pandemic, Glioblastoma, Cerebral metastasis, Tumor volume

## Abstract

The COVID-19 pandemic has disrupted healthcare delivery worldwide, leading to significant delays in cancer diagnosis and treatment. This study aimed to investigate the impact of the pandemic on the diagnosis and treatment of malignant brain tumors, specifically glioblastoma (GBM) and cerebral metastasis (CM), in a specialized neuro-oncology center. We analyzed data from 236 patients diagnosed with previously unknown malignant brain tumors between January 2018 and December 2021. Patients were classified into two groups: pre-COVID (January 2018 to December 2019) and COVID (January 2020 to December 2021). Tumor volumes were compared between the two groups and factors affecting tumor volumes were studied. Of 236 patients diagnosed with previously unknown malignant brain tumors, 114 were in the pre-COVID group and 122 were in the COVID group. Median tumor volumes at first diagnosis were significantly larger in the COVID group compared to the pre-COVID group (21.7 vs 15.7 cm^3^; *p* < 0.05). The survival times for the overall cohort and the GBM and CM subgroups did not differ significantly between the pre-COVID and COVID periods. Delays in diagnosis and treatment during the COVID-19 pandemic led to larger tumor volumes at diagnosis for patients with malignant brain tumors. However, these larger tumors did not result in worse survival outcomes. This counterintuitive finding highlights the crucial role of specialized neuro-oncological centers in mitigating the potential negative impact of delayed treatment and emphasizes the need for continued access to specialized care during times of crisis.

## Introduction

COVID-19 has affected the world in unprecedented ways, resulting in a global pandemic that has severely impacted healthcare systems worldwide. Germany was no exception, implementing a range of measures to reduce the transmission of the SARS-CoV-2 virus [1, 2]. This included excluding patients with even mild flu-like symptoms from non-emergency outpatient care and encouraging the public to avoid contributing to the extreme pressures on healthcare facilities by staying at home and reducing social contacts. Such restrictions may have delayed the diagnosis of a wide range of medical conditions and led to a more severe disease manifestation or advanced stage at diagnosis and later treatment initiation [3].

Delayed diagnosis of a range or cancer types and non-malignant conditions as a consequence of the COVID-19 pandemic has already been described [3–5].

This study aims to investigate the impact of COVID-19 in neuro-oncology, specifically the potential delays in diagnosis and treatment of malignant brain tumors, including cerebral metastases (CM) and glioblastoma (GBM). These tumors often present acutely, with new or progressive neurological deficits, symptoms of raised intracranial pressure, or seizures, and require timely diagnosis and treatment. Owing to the aggressive nature of the disease, diagnosis attainment and treatment initiation should be pursued in a timely fashion. Even during the COVID-19 pandemic era, expert consensus has demanded there should be no delay in treating GBM or CM [6]. Delayed treatment of these conditions can result in more severe disease manifestation or advanced stage at diagnosis and later treatment initiation.

Previous research has already highlighted the delayed diagnosis of various types of cancer and non-malignant conditions as a consequence of the pandemic [7–10]. Here, we aimed at contributing to a better understanding of the impact of the COVID-19 pandemic measures on neuro-oncology, and we highlight the importance of ensuring timely diagnosis and treatment for these conditions, even during a pandemic.

## Materials and methods

### Study design, inclusion, and exclusion criteria

We conducted a retrospective study of patients with an initial presentation and first diagnosis of an intraaxial brain tumor between January 2018 and December 2021 who subsequently underwent biopsy and/or tumor resection. The study design followed the current STROBE criteria guidelines [11]. Tumors had to be neuropathologically confirmed either as isocitrate dehydrogenase (IDH)-wildtype glioblastoma, WHO grade 4, or as a CM [12]. We excluded patients with any diagnosis other than CM or GBM, IDH-wt, and WHO grade 4, according to the current WHO classification. Patients with recurrent, previously known, or progressive tumors were also excluded, as well as those who were unable to undergo MRI examinations. We stratified patients based on the time period of normal, unlimited access to medical care (years 2018–2019, pre-COVID group), and a period with limited access to healthcare due to the SARS-CoV-2 pandemic (years 2020–2021, COVID group).

### Neuropathological assessment

Histopathological and molecular assessment of tumor tissue was performed by an independent, experienced neuropathologist. Tumors were classified according to the then-current World Health Organization grading system for classification of central nervous system tumors, including WHO grade 4, IDH wildtype, and GBM [12]. We excluded gliosarcomas and WHO grades 1–3 glial tumors. Patients with newly diagnosed CM were included, and the origin and type of the primary tumors were recorded. Patients with more than one primary tumor but with a new manifestation of CM were also included, as well as those with a cancer of unknown primary syndrome.

### Treatment pathway

Patients presenting with a cerebral imaging presenting a brain lesion were discussed extensively in our interdisciplinary tumor conference, with input from specialists in all concerned disciplines. Prior to any surgical approach, patients underwent cerebral imaging, including a brain MRI with T1-weighted, T2-weighted, and gadolinium-enhanced sequences. Treatment decisions were made based on contributions from different specialists, with a focus on the most appropriate treatment for the patient. In most cases, the recommended treatment modality was surgical resection or biopsy. Patient preferences and expectations were taken into account in the decision-making process.

After performing a surgical procedure (resection or biopsy), the samples were stored and examined. The histopathology report was discussed by the interdisciplinary tumor board. Standard care for malignant brain tumors involved the combination of ionizing radiation and/or adjuvant chemotherapy, based on patient clinical performance assessed according to the Karnofsky performance scale (KPS) index [13].

Standard adjuvant treatment for patients with GBM was concomitant TMZ-based radio-chemotherapy followed by adjuvant chemotherapy according to the EORTC-26981 trial [13]. Radiation was fractionated up to 60 Gy, and reduced dose regimens were offered in elderly patients according to Perry et al. [14]. Patients with CM received either adjuvant stereotactic radiotherapy to the resection cavity or whole brain irradiation as described previously [15].

Patients underwent regular follow-up with clinical examination and radiological control with a brain MRI, including T1-weighted, T2-weighted, and gadolinium-enhanced sequences. We followed the RANO criteria for assessing glioma progression and the response to treatment, with patients undergoing regular follow-up visits in the outpatient clinic of our neuro-oncological center as proposed by clinical guidelines [16].

### Data collection

Demographic data, age, gender, and surgical and adjuvant therapy information were collected retrospectively from digitalized charts. Pre- and postoperative MRI images were obtained from digital records. Data needed for conducting survival analysis was obtained from our central neuro-oncology database, as well as from patients’ family practitioners.

### Outcome variables

The outcome variables in this study were calculated based on initial MRI examinations. These examinations included T1-weighted, T2-weighted, and gadolinium T1-weighted enhanced sequences, which were transferred to a planning station for analysis using the Brainlab Elements software (Brainlab, Munich, Germany). Contrast-enhancing tumor volumes were calculated using semi-automatic tumor segmentation methods to ensure consistency across all patients. For the calculation of the tumor volume, we used the preoperative MRI images which led to the diagnosis of cerebral tumor.

To reduce the impact of inter-observer variation, all tumor segmentations were performed by a single author.

### Statistic and ethical considerations

For statistical analyses, we used commercially available software (GraphPad Prism version 9 for Windows, GraphPad Software, San Diego, CA, USA). Categorical variables between groups were assessed using Fisher’s exact test. Continuous variables were compared using parametric or non-parametric tests, when appropriate, after testing for Gaussian distribution. Survival analysis was conducted using Kaplan–Meier estimates and the Log-rank (Mantel-Cox) test to estimate overall survival. Univariate (UVA) and multivariate (MVA) Cox regression analyses (Cox proportional hazards survival regression) were then performed to identify predictors of overall survival. *P* values ≤ 0.05 were considered statistically significant.

This study was approved by our local ethics committee (protocol ID number: 2022–2647-Daten). All patients provided written informed consent prior to any diagnostic or therapeutic procedures, and all procedures were performed in accordance with the ethical standards of the institutional research committee and with the 1964 Helsinki Declaration and its later amendments. We also ensured that all patient data was kept confidential and secure, and only used for the purpose of this study. Patient identifiers were removed from the dataset before analysis to maintain anonymity.

## Results

We analyzed data of 236 patients newly diagnosed with brain tumors (113 male, 103 female), with 125 in the pre-COVID group and 111 patients in the COVID group. In total, there were 115 patients with GBM (58 patients in the pre-COVID, 57 patients in the COVID group), and 121 patients with CM (67 patients in the pre-COVID, 54 patients in the COVID group). The median age across the CM and GBM groups was 65 years (range: 33–85 years). The female-to-male ratio was 103:133. The majority of patients (*n* = 171, 72%) underwent tumor resection, whereas 65 (28%) patients had a cerebral biopsy. Two hundred twenty-four patients (95%) had a KPS ≥ 70% or more (24 patients KPS 70%, 57 patients KPS 80%, 95 patients KPS 90%, 48 patients KPS 100%), and 12 patients (5%) had KPS < 70%. Patients with adverse clinical performance and KPS < 70% generally received only a biopsy.

Among the GBMs, 74 patients (64%) had a designated methylation of the MGMT-promoter whereas in 41 patients (36%), the MGMT-promoter region was unmethylated.

For patients with CM, the most frequent primary tumor was lung cancer (27%) followed by tumors originating from the gastrointestinal tract (18%).

The smallest tumor volume measured 0.3cm^3^, the largest 111.4cm^3^, whereas the median tumor volume was 17.7cm^3^. One hundred eighty-four patients (78%) received postoperative radiotherapy. Fifty-two patients (22%) did not receive radiotherapy, either because it was not desired by the patient or their representatives or because the patients died before radiotherapy onset. Twenty-two patients did not receive any radiotherapy because of an acute clinical deterioration and death (14 patients in the pre-COVID and 8 patients in the COVID group). Further patients’ details are provided in Table 1.

**Table 1:** Cumulative descriptive statistical analysis

	Patient groups		
	GBM	CM	Total
Total patients	115	121	236
Pre-COVID group	58	67	125
COVID group	57	54	111
Age (years)			
Median	67	65	65
Minimum	33	37	33
Maximum	83	85	85
Gender			
Female	49	54	103
Male	66	67	133
Surgical procedure			
Biopsy	56	9	65
Resection	59	112	171
MGMT promotor status		NA	
Methylated	74		-
Not methylated	41		-
Spread of metastasis	NA		
Only CNS involvement		59	-
CNS and extra-CNS involvement		62	-
Primary tumor			
Lung cancer	NA	32	-
Gastrointestinal tract cancer	NA	22	-
Malignant melanoma	NA	16	-
Breast cancer	NA	9	-
Other (renal, CUP)	NA	31	-
KPS			
KPS ≥ 70%	109	115	224
KPS < 70%	6	6	12
RT			
Yes	90	94	184
No	25	25	52
Tumor volume (cm^3^)			
Median	28.60	13.5	17.7
SD	24.15	18.07	22.14
Maximum	111.4	69.40	111.4
Minimum	0.53	0.30	0.30
Tumor volume (cm^3^), pre-COVID group			
Median	25.65	12.10	
SD	22.78	18.54	
Maximum	83.70	69.40	
Minimum	0.53	0.30	
Tumor volume (cm^3^), COVID group			
Median	31.50	16.80	
SD	25.35	17.46	
Maximum	111.4	62.90	
Minimum	1.10	0.82	

### Pre-COVID vs. COVID eras

We analyzed the complete cohort of 236 patients, consisting of 125 pre-COVID patients and 111 COVID-era patients.

Notably, there were no significant differences between the two groups in terms of median age (65 vs. 66 years) or gender distribution (females to males *n* = 59 vs. 66, and *n* = 44 vs. 67 in the pre-COVID and COVID groups, respectively, *p* > 0.05).

**Fig. 1:**
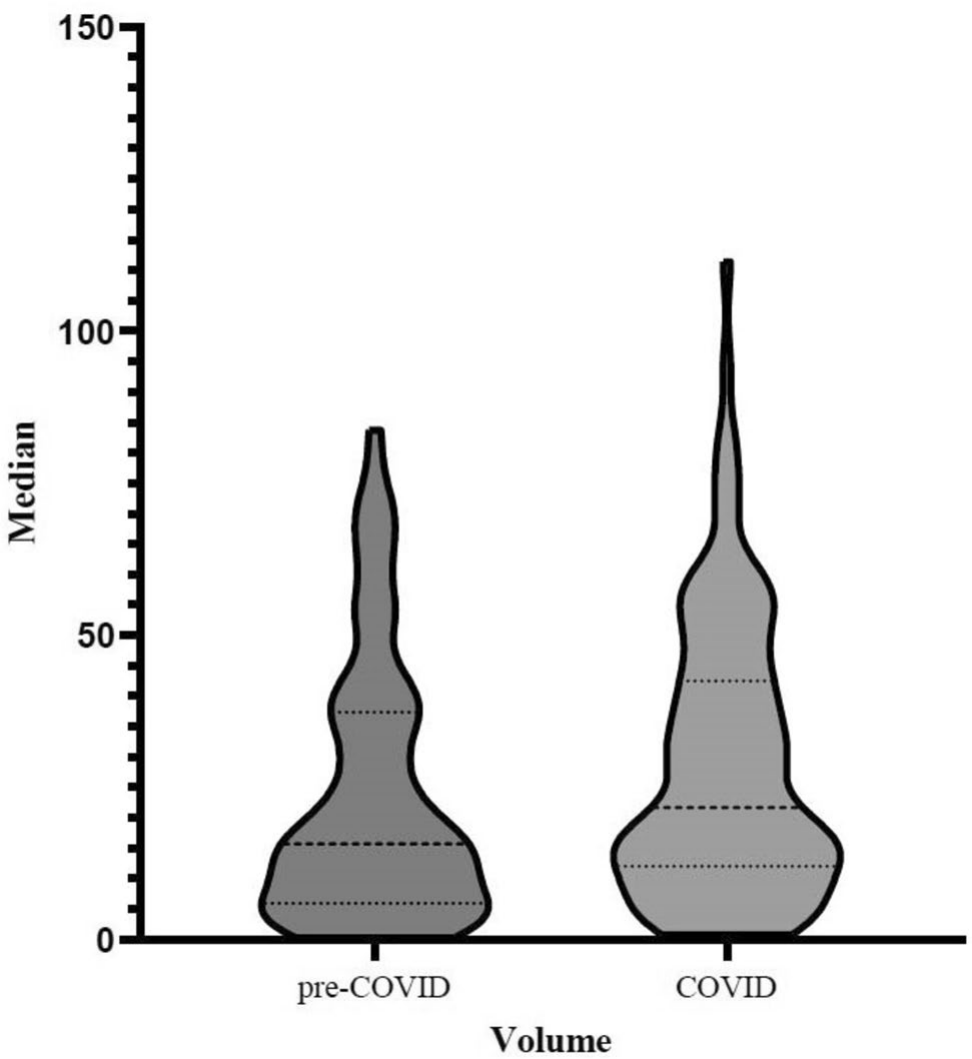
Violin plot comparing tumor volumes (CM and GBM) in the pre-COVID and COVID cohorts. Tumor volumes at time of diagnosis were statistically significantly larger in the COVID group than in the pre-COVID group. The dashed lines represent median values; the dotted lines represent quartiles. The width of the bars corresponds to the number of patients with a given tumor volume

In the pre-COVID group, 119 patients had KPS ≥ 70%, and in the COVID group, 105 patients had KPS ≥ 70% (*p* 0.3363). A biopsy was performed in 38 patients in the pre-COVID group and 27 patients in the COVID group, while surgical resection was performed on 87 and 84 patients, respectively (*p* 0.3108, Fisher’s exact test). The COVID group had statistically significantly larger median volumes (21.70 cm^3^) than the pre-COVID group (15.70 cm^3^, *p* < 0.05, Mann–Whitney *U* test). Regarding radiotherapy, there was no significant difference between the pre-COVID and COVID groups, with 96 and 88 patients receiving treatment, respectively (*p* = 0.648, Mann–Whitney *U* test). Further details are provided in Table 2 and Fig. 1.

**Table 2:** Demographic, performance and treatment parameters and tumor volumes in the pre-COVID and COVID groups

	Pre-COVID group (*n* = 125)	COVID group (*n* = 111)	*p* value
Age			
Median	65	66	0.1950
Minimum	37	33	
Maximum	85	84	
Range	48	51	
Gender			
Female	59	44	0.2439
Male	66	67	
Surgical procedure			
Biopsy	38	27	0.2986
Resection	87	84	
KPS			
≥ 70%	119	105	0.3363
< 70%	6	6	
RT			
Yes	96	88	0.6481
No	29	23	
Volume (cm^3^)			
Median	15.7	21.7	0.0464*
SD	21.3	22.83	
Maximal	0.3	0.82	
Minimum	83.7	111.4	

*KPS* Karnofsky performance status, *RT* radiotherapy, *SD* standard deviation, *Mann–Whitney *U* test for non-parametric variables.

#### GBM

We treated 115 patients with GBM, comprising 58 patients from the pre-COVID group and 57 from the COVID group. The median age of patients was 67 years, with a range from 33 to 83 years, and there were 49 females and 66 males. A biopsy was performed in 56 patients (49%), while 59 (51%) underwent tumor resection. Of the entire cohort, complete resection was achieved in 27 cases (23%), as assessed by postoperative imaging. In the remaining 88 patients (77%), residual tumor was present due to incomplete resection or because only a biopsy was performed. Notably, there were no significant differences in the MGMT promoter status between the pre-COVID and COVID groups. Of the 115 patients, 74 (34 pre-COVID, 40 COVID) had a methylated MGMT promoter and 41 (24 pre-COVID, 17 COVID) had an unmethylated MGMT promoter. The majority of patients (*n* = 109) had a KPS ≥ 70%, with only 6 patients having a KPS < 70%. KPS was also evaluated during follow-up in the majority of patients. KPS at first follow-up, 3 months after the initial surgical procedure, was ≥ 70% in 59 patients (81%), while in 14 (19%), it was < 70. Of the 90 patients who received radiotherapy after surgery (78%), 43 received a standard dose of 60 Gy, while 47 received a lesser dose. Regarding tumor volume, the mean tumor volume measured 32.4 cm^3^, and the median was 28.6 cm^3^ (range 0.5–111.4 cm^3^) for the entire cohort. In the pre-COVID group, median tumor volume was 25.6 cm^3^, which was smaller than in the COVID group with a median of 31.5 cm^3^ (*p* = 0.21, Mann–Whitney *U* test; Table 1).

#### CM

In total, 121 patients with CM were included, with 67 patients in the pre-COVID group and 54 patients in the COVID group. The median age was 65 years (range: 37–85 years), and the female-to-male ratio was 54:67. The majority of patients, *n* = 112 (92.6%), underwent tumor resection, whereas only 9 patients received a biopsy. Seventy-eight patients (64%) had a previously diagnosed malignant neoplasm for which periodic follow-up was conducted, without previously identified CNS involvement, whereas for 43 patients (36%), the CM was the first manifestation of malignancy (25 in the pre-COVID, 18 in the COVID group). In 59 patients (49%), the cerebral metastasis was the sole non-primary-organ involvement, whereas in 62 patients (51%), there was also extra-CNS metastasis. Lung cancer was the most common primary tumor (32 cases), followed by tumors of the gastrointestinal tract (22 cases; 10 esophageal, 10 colorectal, 1 pancreatic, 1 peritoneal). There were also 16 patients with malignant melanoma, 11 patients with renal tumors, and 9 patients with breast cancer. Lastly, 20 patients were classified as having cancers of unknown primary (CUP) despite full investigation.

At presentation, 115 patients had a KPS ≥ 70%, whereas 6 patients had a KPS < 70%. Concerning adjuvant radiotherapy, 94 patients received radiation, and 25 patients did not have any adjuvant therapy. Of those receiving radiation, 47 patients received a dose of 30 Gy, 42 patients had > 30 Gy, and 5 patients had < 30 Gy with the goal of palliation. Radiotherapy began a median of 33 days after the surgical procedure (range: 8–100 days). Details are provided in Table 1.

The mean volume of CM was 19.7 ± 18.1 cm^3^ (median: 13.5 cm^3^, range 0.3–69.4 cm^3^). The tumor volumes in the COVID group were larger with a median of 16.80cm^3^ compared to 12.10cm^3^ in the pre-COVID group (*p* = 0.11, Mann–Whitney *U* test).

### Survival analysis

**Fig. 2:**
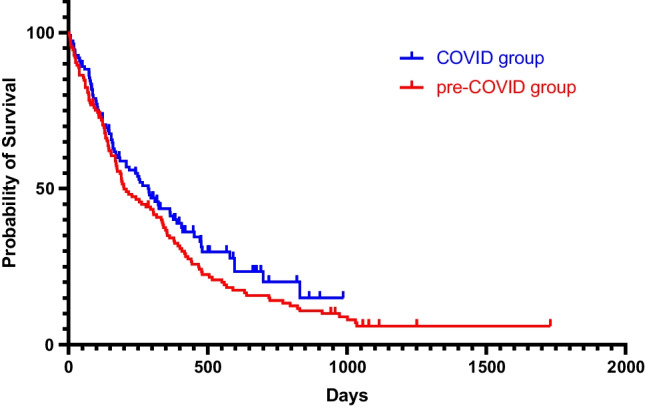
Kaplan–Meier curve depicting the survival time in the pre-COVID (red curve) and in the COVID group (blue curve)

**Fig. 3:**
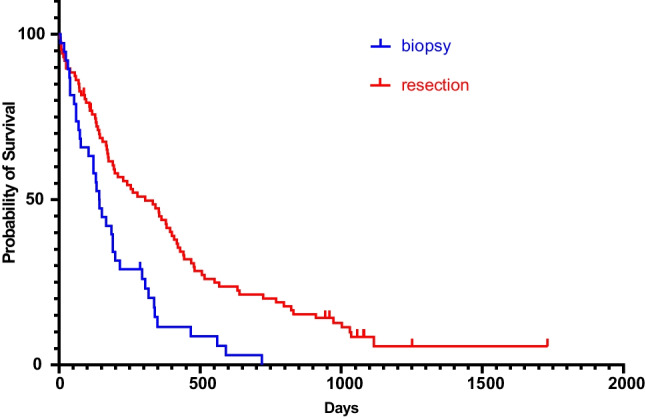
Kaplan–Meier curve depicting the survival time of GBM and CM patients who underwent a biopsy (blue curve) and those who underwent surgical resection (red curve)

**Fig. 4:**
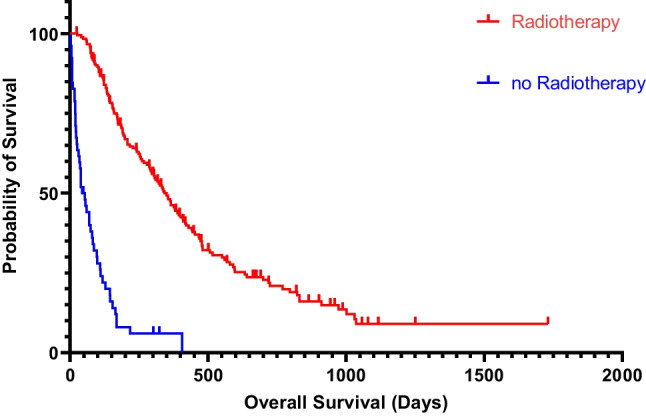
Kaplan–Meier curve depicting the survival time of GBM and CM patients receiving radiotherapy (red curve) and patients without radiotherapy (blue curve), including the 95% CI

All patients were followed up clinically with a median follow-up time of 310 days. The median survival time for the entire cohort (GBM and CM) was 248 days (Figs. 2 and 3). Out of the 65 patients who underwent biopsy, the median survival time was 159 days (95% CI 0.38–0.70). Patients who had surgical resection (*n* = 171) had a longer median survival time of 310 days (95% CI 1.43–2.66), which was statistically significant (*p* = 0.0002, Log-rank test). Patients who received adjuvant radiotherapy (*n* = 184) had a median survival time of 342 days (95% CI 0.10–0.20), which was statistically significant compared to those patients without radiotherapy (*p* < 0.0001, Fig. 4). In the pre-COVID group, the median survival time was 199 days (95% CI 0.52–0.93), compared to 287 days (95% CI 1.08–1.93) in the COVID group, which was not statistically significant (*p* = 0.177, Log-rank test).

Analysis of GBM alone revealed no statistically significant difference in survival times between the pre-COVID and COVID groups (median: 208 vs. 325 days, *p* = 0.108, Log-rank test). However, radiotherapy had a significant impact on GBM survival time, with patients who received radiation having a median survival time of 376 days (95% CI 6.01–15.46) compared to 39 days (95% CI 0.065–0.17) for those who did not receive radiation (*p* < 0.0001, Log-rank test). Additionally we found out that patients underwent GTR had a longer survival (597 days, 95% CI 1.633–4.679) compared to patients with either biopsy or STR (216 days, 95% CI 0.2137–0.6125). This difference in survival time was statistical significant (*p* 0.0028, Log-rank test). The rate of GTR was higher in the COVID group compared to the pre-COVID group (17 of 57 patients vs. 10 of 58 patients, *p* 0.128).

In the cohort with CM, the median survival was 198 days (HR 0.952) in the pre-COVID group and 208 days (HR 1.051) in the COVID group. However, this difference in survival did not reach statistical significance (*p* = 0.8044). Radiotherapy also had a significant impact on the survival time of patients with CM, with those who received adjuvant radiotherapy having a median survival time of 295 days (95% CI 2.73–6.71) compared to 69 days (95% CI 0.15–0.37) for those without radiation (*p* < 0.0001). Table 3 summarizes these findings.

**Table 3:** Survival analysis using the Kaplan–Meier method and Log-rank (Mantel-Cox) test

	Overall survival (days)									
	Total population		GBM		CM		Pre-COVID group		COVID group	
		*p*-values		*p*-values		*p*-values		*p*-values		*p*-values
Cohort										
Pre-COVID group	199	ns (0.1768)	208	ns (0.1075)	198	ns (0.8044)				
COVID group	287		325		208					
Surgical procedure										
Biopsy	159	0.0002	154	< 0.0001	168	ns (0.4440)	143	0.0002	185	ns (0.1854)
Resection	310		417		208		306		310	
MGMT promoter status										
Methylated	─		310	ns (0.2698)	─		─		─	
Not methylated	─		296		─		─		─	
Spread of metastasis										
Only CNS involvement	─		─		355	0.0064	─		─	
Extra CNS involvement	─		─		138		─		─	
KPS										
KPS ≥ 70	248	ns (0.6596)	296	0.4425	208	ns (0.1222)	208	ns (0.6748)	267	ns (0.7314)
KPS < 70	234		520		93		92		387	
RT										
Yes	342	< 0.0001	376	< 0.0001	295	< 0.0001	339	0.292	310	< 0.0001
No	49		39		69		198		80	

Uni- and multivariate Cox regression analyses to evaluate the influence of different predictor variables on survival time were performed. The results of the univariate analysis showed that older age (HR 1.035, *p* < 0.001), male gender (HR 1.425, *p* = 0.017), and failure to receive radiotherapy (HR 7.071, *p* < 0.001) were negatively associated with survival time. On the other hand, a better KPS index (HR 0.982, *p* < 0.001) and tumor resection (HR 0.573, *p* < 0.001) were associated with improved survival time. The results of a multivariable Cox-regression analysis revealed that undergoing a tumor resection (HR 0.423, *p* < 0.001), being younger (hazard ratio 1.040, *p* < 0.001), and receiving radiotherapy (HR 7.626, *p* < 0.001) were statistically significant, independent predictors of improved survival time. Gender and KPS index were not found to be significant predictors.

## Discussion

The present study provides important insights into the impact of the COVID-19 pandemic on the diagnosis and treatment of brain tumors. Our results show that there were no significant differences in the demographic characteristics of patients with brain tumors between the pre-COVID and COVID groups. However, we observed a statistically significant increase in the median tumor volume in the COVID group compared to the pre-COVID group. This suggests that delays in the diagnostic process have led to patients presenting with larger brain tumors, which can negatively affect treatment planning and prognosis.

Pandemic-related delays in seeking medical attention and starting anti-tumor treatment have been identified in a range of diseases and across medical specialties [17]. The impact of these delays has already been widely reported, with underdiagnosis and more advanced stages of tumors, for example, in malignant melanoma, with greater Breslow thickness and higher mitotic activity [4, 9, 18]. Similarly, laryngeal cancer and oral squamosa cell carcinomas have been found to present with significantly larger tumor volumes and more advanced stages [10, 19]. In the UK, there has been an estimated increase in mortality among oncological patients with breast, colorectal, lung, and esophageal cancer [6]. Moreover, non-malignant yet potentially life-threatening conditions such as acute myocardial infarction and pediatric type 1 diabetes have also been presenting and receiving initial management later, leading to more severe and acute cases [3, 20].

Previously, several reports have shown that not only residual tumor volume but also preoperative tumor volume is a negative prognostic factor in gliomas, particularly low grade tumors [21, 22]. For GBM, such a relationship is less well established. While undoubtedly, residual contrast enhancing tissue conveys a negative prognostic influence [23–25], only few groups have yet reported that preoperative tumor volumes might likewise affect survival. Altieri et al. [26] found that not only GTR but also a preoperative tumor volume > 31.35 cm^3^ is a predictor of survival.

The results of our study show that despite larger tumor volumes at diagnosis during the COVID-19 pandemic, the survival times for neuro-oncological patients with GBM or CM did not differ significantly from pre-pandemic times. This counterintuitive finding may be explained by several factors. Larger tumors at diagnosis may indicate a more advanced disease stage. These tumors may not be so easily removed completely, and their treatment poses a challenge even for experienced tumor surgeons [27].

Yet, we were able to achieve high rates of GTR (even higher than in the pre-COVID group) suggesting that expertise in neuro-oncology using multimodal preoperative planning and intraoperative neuro-monitoring is crucial. Examining the two periods, during the pandemic, 30% of patients underwent GTR, whereas in the pre-COVID group, GTR was achieved in only 17%. In part, this may be explained by a change in departmental structure and thus surgical armamentarium. Although the difference in GTR rates was not statistically significant, our results show a tendency to a more radical treatment during the pandemic; in that respect, we also confirmed the association between GTR and survival in our patient cohort. GTR revealed to have a beneficial role in terms of overall survival without higher rates of permanent deficits.

When patients were referred to us during the pandemic, we prioritized malignant tumor cases over patients with benign tumors or degenerative diseases based on the severity of the condition in order to provide optimal treatment. We yet strived to provide tailored, advanced, and personalized care, thus leading to comparable outcomes for patients with larger or more complex tumors. Postoperative treatment during the pandemic did not differ from pre-pandemic times, and the percentage of patients receiving adjuvant treatment and the treatment strategies (Stupp regimen, short-course fractionated radiotherapy for elderly patients) was comparable in both groups.

Another possible reason for the lack of difference in survival outcomes could be the use of telemedicine and remote consultations during the pandemic. The use of telemedical care may have enabled patients to receive earlier or more frequent consultations with their clinicians during adjuvant treatment, leading to better management of symptoms and faster intervention in case of complications. Additionally, the adoption of remote consultations may have increased access to care for patients who live far away from their treating oncology centers. Overall, our findings emphasize the importance of maintaining access to specialized care and telemedicine during the pandemic and beyond, as they can be vital tools in improving care and outcomes for neuro-oncological patients.

It is worth to mention that for all neuro-oncological patients, one of the main goals is maintaining quality of life. Supportive neurorehabilitation, physical therapy, ergo- and logotherapy, and psychosocial care are all structures that support neuro-oncological patients and should be easily accessible. In our center, all patients are supported from our oncology social workers and every cancer patient receives consultation and gets informed for the different supportive facilities. During hospital stay, each patient receives physio-, ergo-, and logotherapy. In cases it was needed, patients could continue the therapy also after hospital discharge. Palliative care was also provided in in- and/or outpatient setting. Supportive care was offered also during the pandemic, and neuro-oncological patients had priority to access those facilities, but always with respect to the measures against COVID-19. There were no noticeable differences in the availability of supportive care measures for our patients, since we strived to uphold treatment for patients with malignant diseases.

The study’s limitations include its single-center nature and the lack of a granular analysis of the diagnostic pathway. Future multicenter analyses are needed to better understand the causes of delays in presentation, diagnosis, and treatment during the pandemic. Additionally, it would be useful to analyze the differential effects of the pandemic at different stages, when varying restriction measures and vaccine implementation were present.

In summary, the study’s findings suggest that delays in the diagnosis of brain tumors during the pandemic have led to larger tumor volumes at initial presentation. However, larger tumors did not result in worse survival outcomes in our series, possibly due to referral to a specialized neuro-oncological centers. In a recent publication, treatment delays for glioblastoma patients during the pandemic in other German centers were also reported, without negatively affecting overall survival [28]. In the latter study, however, tumor volumes were not assessed in detail.

Future research is needed to better understand the causes and effects of delays in diagnosis and treatment during the pandemic.

## Data Availability

Data are available upon request.
